# Prevalence of Cardiovascular Diseases in COPD–OSA Overlap Syndrome Versus COPD Alone: A Systematic Review

**DOI:** 10.1155/pm/2311043

**Published:** 2026-09-12

**Authors:** Ștefania-Florina Oprea, Ștefan Dumitrache-Rujinski, Camelia Cristina Diaconu

**Affiliations:** ^1^ Faculty of Medicine, Carol Davila University of Medicine and Pharmacy, Bucharest, Romania, umfcaroldavila.ro; ^2^ Pneumology IV Department, Marius Nasta Institute of Pulmonology, Bucharest, Romania; ^3^ Internal Medicine Department, Clinical Emergency Hospital of Bucharest, Bucharest, Romania, scub.ro; ^4^ Academy of Romanian Scientists, Bucharest, Romania, aos.ro

**Keywords:** cardiovascular disease, COPD, OSA, overlap syndrome

## Abstract

**Background:**

Chronic obstructive pulmonary disease (COPD) is frequently associated with cardiovascular comorbidities. Obstructive sleep apnea (OSA) may coexist with COPD, forming the so‐called overlap syndrome (OS), and may further increase cardiovascular risk through intermittent hypoxia, systemic inflammation, and sympathetic activation. This systematic review is aimed at synthesizing and comparing the burden of cardiovascular comorbidities in patients with COPD and coexisting OSA versus those with COPD alone, thereby identifying the additional cardiovascular burden associated with OSA and gaps in current knowledge.

**Materials and Methods:**

A systematic search was conducted in PubMed and Web of Science from inception to 3^rd^ of January 2026, using predefined search terms related to COPD, OSA, and cardiovascular diseases. Only observational studies in adults were included.

**Results:**

Nineteen studies were included in the final analysis. Most studies had a cross‐sectional or retrospective cohort design. Cardiovascular disease was reported as a baseline characteristic in nearly all included studies; however, none evaluated the prevalence of cardiovascular disease as a primary outcome; these conditions were reported mainly as associated comorbidities. Overall, most included studies reported a higher prevalence of cardiovascular diseases—particularly systemic arterial hypertension—in patients with overlap syndrome compared with COPD alone, frequently reaching statistical significance; however, this finding derived from unadjusted comparisons of baseline comorbidities, and no study adjusted for confounders such as age, body mass index, or smoking.

**Conclusions:**

Although many of the observed differences were statistically significant, they derived from unadjusted comparisons of baseline characteristics, which limits the ability to draw firm conclusions regarding causality. Future studies including larger populations of patients with COPD and OSA are required to accurately determine the prevalence of cardiovascular disease and better describe the relationship between COPD, OSA, and cardiovascular comorbidities.

## 1. Introduction

With approximately 400 million people affected, chronic obstructive pulmonary disease (COPD) is one of the leading causes of death worldwide, ranking third overall [1]. Obstructive sleep apnea (OSA) affects around 936 million individuals globally and represents a prevalent respiratory disorder [2].

The association between COPD and OSA, known as overlap syndrome (OS), was first described by Flenley in 1985 [3], highlighting more severe clinical consequences of the combined syndrome compared with each condition alone. A recent meta‐analysis including over 1.8 million subjects reported a prevalence of OS of approximately 28.3% in selected populations [4], which appears to be increasing compared with prevalence estimates of 1% to 3.6% reported in 2015 [5]. This may reflect differences in study methodology and population characteristics rather than a true increase. By contrast, the prevalence of OSA among patients with COPD varies widely, between 10% and 78% [6], reflecting substantial heterogeneity in study cohorts, geographic regions, and potential selection bias.

The association between COPD and OSA is complex; the two conditions are interconnected and exert a synergistic effect on systemic inflammation, sympathetic activation, intermittent hypoxemia, and oxidative stress [7]. These pathophysiological mechanisms predispose to and may exacerbate cardiovascular comorbidities. The main cardiovascular diseases associated with both conditions include arterial hypertension, heart failure, cardiac arrhythmias, and cerebrovascular diseases such as stroke, given the presence of multiple shared risk factors. Age, male sex, smoking, as well as socioeconomic and environmental factors—such as limited access to healthcare, occupational exposure, and air pollution—are common risk factors that contribute to the development or worsening of all three conditions.

This systematic review is aimed at synthesizing and comparing the burden of cardiovascular diseases in patients with COPD and coexisting OSA versus those with COPD alone, thereby identifying the gaps in current knowledge.

## 2. Materials and Methods

### 2.1. Protocol

A systematic literature search was conducted to identify and compare the prevalence of cardiovascular diseases in patients with COPD and coexisting OSA versus those with COPD alone. This review was conducted and reported in accordance with the 2020 guidelines of the Preferred Reporting Items for Systematic Reviews and Meta‐Analyses (PRISMA) [8]. A review protocol was developed a priori, before the initiation of the literature search and data analysis, specifying the study objectives, eligibility criteria, search strategy, and methods for data extraction and synthesis. The protocol was not registered in a public database, given the descriptive and exploratory nature of the review. The full PRISMA 2020 checklist is available in the (Table S1).

### 2.2. Eligibility Criteria

Eligibility followed a PECO framework. Population: Adults (≥ 18 years) with COPD (according to accepted international guidelines (e.g., GOLD) or ICD‐9, ICD‐10) and OSA (based on respiratory polygraphy (RP), home sleep apnea testing (HSAT), or polysomnography, and ICD‐9, ICD‐10). Given the variability in terminology, both “RP” and “HSAT” were considered eligible when the recorded parameters corresponded to Type III sleep monitoring devices. Studies of mixed respiratory populations in which COPD data could not be extracted separately, and patients with central sleep apnea or sleep‐related hypoventilation without OSA, were excluded. Intervention or exposure: This systematic review examines OSA as an exposure in adults with COPD. Comparator: COPD alone was selected as the comparator group because this review aimed at describing and comparing the burden of cardiovascular disease observed in patients with OS relative to patients with COPD alone. A comparison against OSA alone addresses a different question and is proposed as a future direction of research. Study design: The eligible studies included observational studies, such as cross‐sectional studies and cohort studies, both prospective and retrospective. Randomized controlled trials, case reports, case series, narrative reviews, systematic reviews, conference abstracts, editorials, letters, and animal studies were excluded. Randomized controlled trials were not eligible because the review addresses the prevalence of cardiovascular comorbidities—an epidemiological question for which observational designs provide representative, real‐world estimates. Outcomes: The main outcome of this review is to assess the prevalence of cardiovascular diseases in adults with COPD and coexisting OSA (OS), compared with adults with COPD alone. Cardiovascular diseases include hypertension, coronary artery disease, heart failure, cardiac arrhythmias (including atrial fibrillation), and cerebrovascular disease. Prevalence was extracted as reported proportions or percentages at the time points assessed in each study. Additional outcomes include the prevalence of each cardiovascular disease, reported separately in patients with OS and in patients with COPD alone. Language: The review includes only studies published in English. Publication date: Studies indexed up to the final search (3^rd^ of January 2026) were eligible, so no restrictions by year of publication were made.

### 2.3. Search Strategy

The search was performed across two electronic databases, PubMed and Web of Science, from inception to the 3^rd^ of January 2026, using predefined search terms related to COPD, OSA, and cardiovascular diseases.

Two complementary search strategies were applied in each database. These two databases were selected for their multidisciplinary coverage of the respiratory and cardiovascular literature, with robust citation indexing. The primary search strategy focused on identifying studies of COPD–OSA overlap syndrome, irrespective of outcomes, to maximize sensitivity. Cardiovascular outcomes were subsequently extracted from full‐text articles. A secondary search including cardiovascular‐related terms was conducted to identify additional studies specifically addressing cardiovascular comorbidities. Results were combined and duplicates removed before screening.

For transparency and reproducibility, the complete search strings used for both primary and secondary search are mentioned in the supporting information section (Table S2).

### 2.4. Selection Process and Data Collection

**Figure 1:**
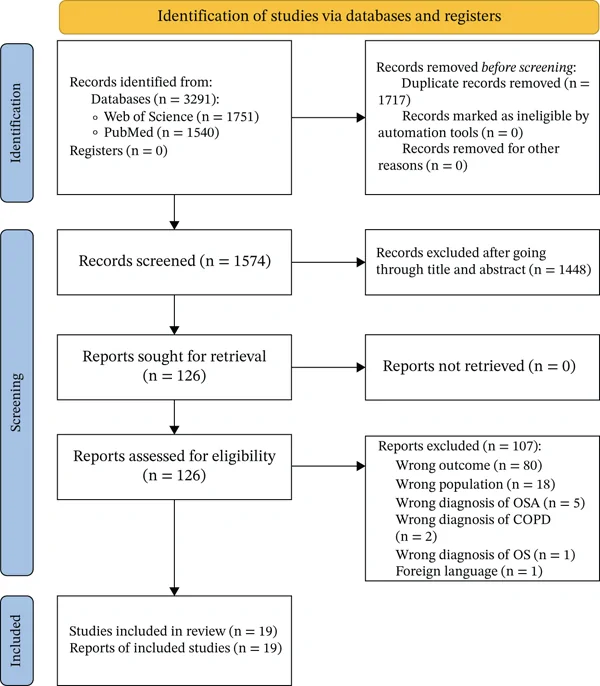
PRISMA 2020 flow diagram of the study selection process. Adapted from Page et al. [8] (Licensed under CC BY 4.0).

The search process and study selection were performed independently by two researchers, based on predefined and strictly applied eligibility criteria to minimize selection bias. Records from both databases were imported into Rayyan (Rayyan Systems Inc., Cambridge, Massachusetts United States). Duplicate records were identified by the automated algorithm and confirmed manually. Irrelevant articles were excluded based on title and abstract screening. The reports assessed for eligibility were evaluated using the inclusion and exclusion criteria. The selection process is documented in a PRISMA 2020 flow diagram (Figure 1).

Extracted data included first author, year of publication, country, study design, data source, total sample size, COPD and OSA (OS) sample size, COPD alone sample size, mean age, male percentage, mean body mass index (BMI), OSA definition, hypertension, coronary artery disease, heart failure, atrial fibrillation, stroke/cerebrovascular diseases, adjusted analysis, notes, and results. The header row of the extraction table is attached in the supporting information section (Table S3). Missing data were recorded as “not reported” (NR), and no assumptions were made beyond the reported information.

### 2.5. Risk of Bias Assessment

The risk of bias was assessed using the Newcastle–Ottawa Scale (NOS). A modified NOS scale for cross‐sectional studies was used, with a maximum of 10 points. Studies scoring 7–9 points (7–10 points on the modified NOS scale) were considered high quality, 5–6 moderate quality, and < 5 low quality. The NOS scores for each study are presented in Table S4 from the supporting information section. Disagreements between reviewers were resolved through discussion and consensus.

### 2.6. Synthesis Methods

The effect measure used was the prevalence of cardiovascular diseases, expressed as proportions or percentages as reported in the included studies.

A quantitative meta‐analysis was not performed because of substantial clinical and methodological heterogeneity across studies, including differences in OSA and COPD definitions, cardiovascular outcome assessment, study design, and reporting of effect estimates. Most studies reported cardiovascular diseases as baseline comorbidities rather than as standardized outcomes, precluding meaningful statistical pooling. A narrative synthesis was performed, structured by each cardiovascular disease. For the same reasons, no sensitivity analysis, formal reporting‐bias assessment, or certainty‐of‐evidence (e.g., GRADE) rating was undertaken.

## 3. Results

### 3.1. Study Selection and Screening

The initial search identified 3291 studies, of which 1717 were excluded because of duplication. After a first rigorous screening of titles and abstracts, 1448 records were excluded. One hundred twenty‐six articles were considered potentially relevant according to the inclusion and exclusion criteria. An additional 10*7* articles were excluded after full‐text review. The reasons for the exclusion at this stage were documented in the PRISMA flow diagram (Figure 1). The detailed list of the exclusion reasons is attached in the supporting information section. (Table S5).

### 3.2. Study Characteristics

Following the selection process, 19 studies published between 2014 and 2025 met the inclusion criteria and were included in the review. The study populations were heterogeneous, with the majority originating from China (11 studies) [9–19]; however, studies involving populations from the United States [20, 21], Spain [22], France [23], Australia [24], Sweden [25], Switzerland [26], and Brazil [27] were also included. Of the included studies, three were retrospective observational studies [13, 14, 24], two were prospective observational studies [22, 23], and the remaining studies were observational with a cross‐sectional design.

Study cohort sizes ranged from 50 [18] to 968 [16] participants; however, in some studies, the initial population was larger because it also included a comparator group consisting of patients with OSA alone, in addition to OS and COPD‐only groups. The study populations were predominantly male, comprising adults with a mean age above 60 years, with one exception in which men accounted for 49.4% of the overlap population and 36.9% of the COPD‐only population, and the mean age was below 60 years [21]. None of the included studies reported cardiovascular disease prevalence as a primary outcome; instead, cardiovascular diseases were described as baseline characteristics.

Detailed population characteristics for each study, as well as the prevalence of cardiovascular diseases, are provided in the supporting information section Table S6. BMI and obesity were recorded as participant characteristics.

OSA was confirmed by polysomnography in 10 studies, by Type III devices (RP/HSAT) in five, by either polysomnography or RP in one, and three studies relied on self‐reported or physician‐diagnosed OSA (one supported by a previous sleep study). The AHI threshold of 15/h was reported in seven studies, 10/h in three, and 5/h in five. In one study, there was a composite criterion of AHI ≥ 5/h with symptoms or ≥ 15/h without symptoms, and in the last three of them, there was no numerical threshold specified. This variability in diagnostic modality and AHI threshold is an important source of heterogeneity and limits the direct comparison between OSA severity.

COPD was defined spirometrically using a postbronchodilator FEV1/FVC < 0.70 in accordance with GOLD criteria in 15 studies, whereas one study relied on self‐report COPD, and the remaining three did not specify the diagnostic criterion. COPD severity (GOLD stage) was reported in 13 studies, another five only the FEV1%, and only one had no severity measurement. Exclusion of patients with asthma was explicitly reported in four studies Zhao [12], Zhu [19], Wang 2019 [18], Wang 2020 [15], generally based on clinical history and/or bronchodilator reversibility; the remaining studies that applied the GOLD criterion of postbronchodilator FEV1/FVC < 0.70, makes the inclusion of patients with classic, fully reversible asthma unlikely, although it does not entirely exclude long‐standing asthma with persistent airflow limitation (asthma–COPD overlap). Also, in the studies based on self‐reported or unspecified diagnostic criteria, the exclusion of asthma could not be ascertained.

### 3.3. Results of Individual Studies

The prevalence of cardiovascular diseases is detailed for each study in Table 1, with statistical significance reported for each condition. Given the heterogeneity of the included studies, cases in which a specific pathology was not reported were marked as NR.

**Table 1:** Results of cardiovascular disease prevalence in the included studies.

Author, year	Cardiovascular diseases, % (overlap syndrome vs. COPD alone)						Notes
	Hypertension	Coronary artery disease	Heart failure	Atrial fibrillation	Stroke/cerebrovascular disease	Cardiovascular disease (composite)	
Marin et al. 2025 [22]	(42.6 vs. 34.5)	(14.0 vs. 10.2)	NR	NR	NR	NR	Includes iNH group; primary outcome all‐cause mortality; CVD reported at baseline.
Coiffier et al. 2024 [23]	(53.7 vs. 30.5) ^∗^	(27.4 vs. 14.3) ^∗^	NR	NR	(8.4 vs. 4.8)	(72.6 vs. 49.5) ^∗^	CVD reported as prevalence, no longitudinal risk estimates.
Nguyen et al. 2024 [24]	(75.0 vs. 50.0) ^∗^	(20.5 vs. 22.6)	(31.8 vs. 21.8)	(25.0 vs. 17.7)	(9.1 vs. 5.6)	NR	Patients with COPD requiring acute NIV, heterogeneous OSA diagnosis
Hansson et al. 2023 [25]	(71.4 vs. 44.6) ^∗^	(24.5 vs. 8.9) ^∗^	(20.4 vs. 7.1) ^∗^	(10.2 vs. 5.4)	(2.0 vs. 16.1) ^∗^	NR	REM‐related OSA subgroup had higher AF prevalence
Zeng et al. 2022 [9]	(47.7 vs. 40.8)	(16.3 vs. 20.3)	NR	NR	(8.0 vs. 6.3)	NR	Primary outcome polycythemia; CVD reported as baseline
Zhang et al. 2022 [10]	(47.9 vs. 29.3) ^∗^	(10.3 vs. 10.3)	NR	NR	NR	NR	Primary outcome predictors and outcomes of OSA. CVD reported as baseline.
Clímaco et al. 2022 [27]	(33.3 vs. 45.1)	NR	NR	NR	NR	NR	Primary outcome pulse wave velocity. Hypertension the only CVD reported.
Tang et al. 2021a [11]	(60.8 vs. 29.7) ^∗^	(25.7 vs. 11.7) ^∗^	(10.8 vs. 1.4) ^∗^	(10.8 vs. 5.4)	(20.3 vs. 10.4) ^∗^	NR	OS group relatively small (*n* = 74). Includes OSA‐alone group
Zhao et al. 2021 [12]	(41.1 vs. 20.8) ^∗^	(13.9 vs. 4.2) ^∗^	NR	NR	NR	NR	Primary outcome anxiety and depression. CVD reported as baseline.
Liu et al. 2021 [13]	(51.7 vs. 47.7)	(45.8 vs. 44.2)	NR	NR	NR	NR	Patients with COPD and acute respiratory failure requiring invasive or noninvasive ventilation
Tang et al. 2021b [14]	(70.9 vs. 28.5)^a^	(30.4 vs. 9.5)^a^	(35.4 vs. 8.9)^a^	(13.9 vs. 4.4)^a^	(19.0 vs. 13.3)	NR	Bonferroni‐corrected threshold applied. CVD reported as baseline.
Wang et al. 2020 [15]	(67.0 vs. 64.9)	(60.4 vs. 44.4) ^∗^	NR	NR	NR	NR	Primary outcome mild cognitive impairment; uncontrolled hypertension and acute left‐sided heart failure excluded.
Hu et al. 2020 [16]	(40.9 vs. 31.8) ^∗^	NR	NR	(2.6 vs. 2.3)	(9.5 vs. 9.1)	(24.4 vs. 21.4)	No significant differences between the two groups in the prevalence of overall cardiovascular disease.
Sun et al. 2019 [17]	(42.9 vs. 46.0)	(23.2 vs. 14.0)	(16.1 vs. 12.0)	NR	(10.7 vs. 6.0)	NR	OS associated with higher PAP and increased risk of pulmonary hypertension. Cerebrovascular reported rather than stroke.
Wang et al. 2019 [18]	(60.0 vs. 36.0) ^∗^	(36.0 vs. 16.0) ^∗^	NR	NR	(20.0 vs. 8)	NR	Primary outcome endothelial function. Includes four groups (overlap, COPD alone, OSA alone, controls).
Zhu et al. 2019 [19]	(42.1 vs. 35.0)	NR	NR	NR	NR	NR	Primary outcome lung function and apnoea–hypopnoea index; hypertension the only CVD reported.
Fitzgibbons et al. 2019 [20]	(72.1 vs. 55.9) ^∗^	(39.3 vs. 25.1) ^∗^	(21.3 vs. 6.7) ^∗^	NR	NR	NR	Primary outcome physical activity. Self‐reported physician diagnosed OSA
Du et al. 2018 [21]	(60.6 vs. 41.1)	NR	NR	NR	NR	(46.0 vs. 21.8)	NHANES 2005–2008; small overlap group (*n* = 90).
Steveling et al. 2014 [26]	(60.6 vs. 37.5) ^∗^	NR	NR	NR	NR	(18.2 vs. 12.5)	Primary outcome predictors of overlap syndrome. Overall CVD prevalence did not differ significantly.

*Note:* Values are the prevalence (%) reported in each study for the OS group and the COPD‐alone group, respectively. Corresponding numerators and denominators are provided in Table S7, and study population characteristics in Table S6. Tang et al. 2021a and Tang et al. 2021b denote two distinct publications by the same group (references [11] and [14], respectively). For Du et al. 2018, values are survey‐weighted prevalence estimates and cannot be derived from the unweighted counts in Table S7; the *p*‐values reported in that study refer to the comparison across all four exposure groups, so no pairwise significance marker is applied.

Abbreviations: AF: atrial fibrillation; COPD: chronic obstructive pulmonary disease; CVD: cardiovascular disease; iNH: isolated nocturnal hypoxia; NIV: non‐invasive ventilation; NHANES: National Health and Nutrition Examination Survey; NR: not reported; OSA: obstructive sleep apnea; OS: overlap syndrome (COPD and OSA); PAP: pulmonary artery pressure; REM: rapid eye movement.

^a^Statistically significant at the Bonferroni‐corrected threshold of *p* < 0.017 applied by Tang et al. 2021b.

^∗^Difference between the overlap syndrome group and the COPD‐alone group statistically significant at *p* < 0.05, as reported in the primary study.

Across studies, hypertension was the most consistently reported cardiovascular disease, whereas coronary artery disease, heart failure, arrhythmias, and stroke were less frequently and inconsistently assessed.

To improve data transparency, a supporting table reporting the number and percentage of cardiovascular diseases in each study and group was included (Table S7).

### 3.4. Synthesis of Results

Across all the included studies, cardiovascular diseases were reported as unadjusted baseline characteristics. No study estimates the difference in cardiovascular prevalence between the overlap‐syndrome and COPD alone groups in a model adjusted for confounders such as age, BMI, or smoking. Some of the studies performed adjusted analyses but focused on other primary outcomes, such as all‐cause mortality or predictors of OSA, rather than on the cardiovascular comparison itself. Consequently, the higher cardiovascular prevalence observed in the overlap group across most studies cannot be separated from confounding by older age and higher BMI, which frequently characterize this group.

#### 3.4.1. Hypertension

Arterial hypertension was reported as a baseline characteristic in all included studies. Among the included studies, 11 out of 19 reported a higher prevalence of hypertension in the OS group compared with COPD alone, while six studies reported numerically more HTN in OS vs COPD with no statistical difference. Two studies [17, 27] reported numerically more HTN patients in COPD, but with no statistical significance. In addition, the study by Wang et al. [15] reported no statistically significant differences between groups; patients with uncontrolled hypertension and acute left‐sided congestive heart failure were excluded.

Across the included studies, the reported prevalence of arterial hypertension ranged from approximately 33.3% to 75% in patients with OS and from 21% to 64% in patients with COPD alone.

#### 3.4.2. Coronary Artery Disease

Data on coronary artery disease were extracted from 14 studies. Eleven of these studies reported a higher prevalence of coronary artery disease in the OS group compared with the COPD‐only group, with eight studies demonstrating statistical significance. Nevertheless, one study reported nearly identical prevalence rates between groups [10]. Two of them reported an even higher number of patients with coronary artery disease in the COPD‐only group [9, 24].

Wang et al. [15] conducted a cross‐sectional study comparing patients with OS and COPD alone. Although the primary outcome was mild cognitive impairment, the study also reported that coronary artery disease and myocardial infarction were more prevalent in the overlap group.

#### 3.4.3. Heart Failure

Heart failure was assessed in only six of the included studies. All six reported a higher prevalence of heart failure in the OS group compared with the COPD‐only group, with four studies demonstrating statistical significance.

Fitzgibbons et al. [20], who compared physical activity levels between patients with OS and those with COPD alone, reported that heart failure and other cardiovascular comorbidities, including hypertension and coronary artery disease, were more prevalent in the overlap group.

#### 3.4.4. Atrial Fibrillation

Atrial fibrillation was reported in five studies, all of which reported a numerically higher prevalence in the OS group compared with the COPD‐only group; however, the statistical significance was reached in only one study (Tang et al. [14]: 13.9% vs. 4.4%, *p* = 0.009).

#### 3.4.5. Cerebrovascular Disease

Cerebrovascular diseases were reported in nine studies, either as cerebrovascular disease or as a history of stroke. Eight studies reported a higher prevalence in the OS group compared with the COPD‐only group; in one of these the difference reached statistical significance (Tang et al. [11] 20.3% vs. 10.4%, *p* = 0.027), whereas the remaining studies did not reach statistical significance. In contrast, Hansson et al. [25] reported that a history of stroke was more prevalent among patients with COPD without OSA, a difference that reached statistical significance (2.0% vs. 16.1%, *p* = 0.015).

#### 3.4.6. Cardiovascular Disease

Four of the included studies [16, 21, 23, 26] reported cardiovascular diseases as a general entity, and all described a higher prevalence in the OS group. The difference reached statistical significance only in Coiffier et al. (72.6% vs. 49.5%) [23]. Du et al. reported survey‐weighted estimates of 46.0% versus 21.8%, but the accompanying *p*‐value refers to the comparison across all four exposure groups rather than to the overlap versus COPD‐alone contrast.

## 4. Discussion

### 4.1. Main Findings

In this systematic review, we synthesized evidence from 19 observational studies, comparing the prevalence of cardiovascular disease in patients with OS (COPD and OSA) versus COPD alone. Most studies reported a higher prevalence of cardiovascular disease, particularly systemic arterial hypertension, in the OS group, and in many studies this difference reached statistical significance (Table 1). The cardiovascular diseases were reported mainly as baseline characteristics, not as a primary outcome. The few studies that performed adjusted analyses were directed at other primary outcomes, such as all‐cause mortality or predictors of OSA. Consequently, despite the consistency and frequent statistical significance of the unadjusted association, the excess cardiovascular burden observed in the overlap group cannot be attributed only to OSA, as it is likely confounded by the older age and higher BMI that characterize these patients. These results should be regarded as hypothesis‐generating rather than as evidence of an independent effect of OSA.

Systemic arterial hypertension was the most consistently reported cardiovascular disease and was significantly more prevalent in the overlap group than in COPD alone in most studies, including the study by Hu et al. [16], which was specifically designed to test this association. This observation is pathophysiologically plausible, given the synergistic effects of chronic and intermittent hypoxemia, sympathetic activation, and systemic inflammation in OS. However, the interpretation of these findings is limited by the fact that hypertension was not evaluated as a primary outcome and was not adjusted for confounding factors, so this consistent association cannot be attributed to OSA independently. Sun et al. [17] reported numerically more HTN in patients with COPD alone as baseline characteristics, with no statistical significance.

Evidence regarding coronary artery disease, heart failure, atrial fibrillation, and cerebrovascular disease was more limited as these conditions were assessed in fewer studies. Where reported, they were generally more prevalent in the overlap group, reaching statistical significance for coronary artery disease and heart failure in several studies, and less consistently for atrial fibrillation and cerebrovascular disease.

Coronary artery disease was the second most frequently reported comorbidity and was more prevalent in the overlap group in most studies, reaching statistical significance in 8 of them (Table 1). Intermittent hypoxemia, oxidative stress, and endothelial dysfunction, together with sympathetic activation, are recognized drivers of atherogenesis, as Wang et al. [18] also reported.

Heart failure was assessed in only six studies and was consistently more prevalent in OS, reaching statistical significance in four of them. Pathophysiological mechanisms that can explain the higher heart‐failure burden are increased right‐ and left‐ventricular afterload through pulmonary vascular remodeling and systemic hypertension due to sustained and intermittent hypoxemia. Additionally, nocturnal negative intrathoracic pressure swings and sympathetic activation increase the myocardial wall stress.

Atrial fibrillation was infrequently assessed and, in some studies, grouped under broader arrhythmia categories, further limiting comparability.

Interestingly, Hansson et al. [25] reported a higher prevalence of atrial fibrillation in patients with REM‐related OSA, which may indicate phenotype‐specific cardiovascular vulnerability, as REM‐related OSA has been associated with greater sympathetic activation and intermittent hypoxemia.

Although they were excluded at the eligibility screening stage, two large retrospective cohort studies reported a higher incidence of atrial fibrillation [28, 29] and malignant arrhythmias [29] in patients with COPD–OSA overlap compared with those with COPD alone, suggesting a potential increase in cardiovascular risk beyond baseline comorbidity burden.

Most of the studies were conducted in East Asian, predominantly male cohorts with comparatively low BMI. It is known that cardiovascular comorbidities differ by ethnicity and sex, so these characteristics limit the generalizability of the findings to females, individuals with a higher BMI, or Western population.

Where COPD severity was reported, no study found OS to be associated with more severe airflow limitation than COPD alone. In the studies that reported FEV1 % predicted without formal GOLD staging, lung function did not differ significantly between the two groups. A significant inverse relationship, overlap being less frequent or associated with milder airflow limitation as COPD severity increased, was demonstrated in six studies with formal GOLD staging (Zhu [19]; Nguyen [24]; Zeng [9]; Hu [16]; Marin [22]; Zhang [10]); for example, the prevalence of overlap fell from 78.6% in GOLD 1 to 58.4% in GOLD 4 [16], and severe airflow limitation independently predicted a lower likelihood of OSA in other studies (Marin [22], OR 0.49; Zhang [10], OR 0.58). This pattern is attributed to a protective effect of emphysema and lung hyperinflation on upper‐airway collapsibility [16, 19], and when examined in multivariable analysis, the apparent relationship was abolished by adjustment for BMI [26]. Taken together, these findings indicate that the higher cardiovascular prevalence observed in overlap groups cannot be explained by greater COPD severity and is most consistent with confounding by obesity.

The BMI was numerically higher in the overlap group across several studies, and statistical comparisons were reported inconsistently. Given that obesity is associated with both OSA and cardiovascular risk, it could be a potential confounder. Nevertheless, a recent population‐based study found that COPD‐OSA overlap was associated with greater subclinical atherosclerosis and arterial stiffness than moderate‐severe OSA alone, even in the nonobese men [30]. This suggests that the excess cardiovascular risk in the OS is not fully explained by obesity alone.

Large prospective community‐based cohorts have established that OSA is independently associated with cardiovascular disease. In the Sleep Heart Health Study, OSA predicted incident heart failure and coronary heart disease in middle‐aged men [31], whereas a sex‐specific analysis of participants in both Atherosclerosis Risk in Communities and Sleep Heart Health studies found OSA assessed in midlife to be independently associated with incident heart failure or death in women, but not in men [32]. More recently, sleep apnea‐specific burdens, the hypoxic and ventilatory burdens, were independently associated with incident cardiovascular disease and mortality in the Multi‐Ethnic Study of Atherosclerosis (MESA) and the Osteoporotic Fractures in Men (MrOS) cohorts. The hypoxic burden was minimally affected by obesity, the risk being attributable to the ventilatory disturbance of OSA rather than obesity [33]. But these landmark cohorts did not specifically compare COPD‐OSA overlap with COPD alone and therefore could not quantify the additional cardiovascular burden attributable to OSA within a COPD population.

The role of continuous positive airway pressure (CPAP) therapy in modifying cardiovascular risk and cardiovascular mortality in OS is increasingly studied. However, the evidence base is still heterogeneous in design. A consistent number of observational OS cohorts showed that greater CPAP use has been associated with reduced mortality. In one of them, patients with untreated OS had significantly more cardiovascular deaths than those treated with CPAP, who showed improved survival and fewer hospitalizations [34]; the same benefit was also reported in relation to CPAP use or adherence in subsequent cohorts [35, 36] and in one systematic review [37]. This evidence, however, is entirely observational. Randomized controlled trials in OSA, rather than an overlap population, as in the SAVE trial, did not demonstrate a reduction in cardiovascular events. No completed randomized trial has evaluated CPAP for cardiovascular outcomes specifically in OS. CPAP treatment was generally not reported in the included studies, and where reported, it was not analyzed as a modifier of the cardiovascular prevalence, so its effect on the cardiovascular prevalence cannot be determined.

### 4.2. Strengths

The strengths of this systematic review include a comprehensive search strategy, application of predefined inclusion and exclusion criteria, and a focused evaluation of cardiovascular disease prevalence in OS. The detailed reporting of individual cardiovascular diseases and the cautious interpretation of heterogeneous observational data further strengthen the validity and clinical relevance of the findings.

A key finding of this review is the lack of studies specifically designed to assess cardiovascular disease prevalence as a primary outcome in OS.

This systematic review is among the few focusing on the prevalence of cardiovascular diseases in the OS group versus COPD alone, as most of the published literature concentrates on comparing OS versus OSA. A previous systematic review and meta‐analysis also examined cardiovascular outcomes in OS versus COPD [38]. The present review extends that work by capturing 11 additional studies published from 2021 onward—including several of the largest and most recent cohorts—that were not previously available. Finally, rather than pooling unadjusted estimates—which risks presenting confounded associations as robust effects—we deliberately adopted a narrative synthesis and critically appraised the unadjusted, baseline‐characteristic nature of the data, underscoring that no study has yet assessed cardiovascular disease as a prespecified, confounder‐adjusted outcome in a three‐group (overlap, COPD‐alone, OSA‐alone) design.

### 4.3. Limitations and Sources of Heterogeneity

This review has several limitations. The principal limitation of the available evidence is that cardiovascular diseases were reported almost exclusively as unadjusted baseline characteristics in predominantly cross‐sectional studies. Consequently, the observed numerical differences cannot separate the contribution of OSA‐related pathophysiology from confounding factors like age, obesity, and smoking, and should be regarded as hypothesis‐generating rather than as estimates of an independent effect. Substantial heterogeneity limited quantitative synthesis. A further limitation concerns the OSA‐only comparator group. In the absence of a group of patients with isolated OSA, the excess prevalence observed in the overlap group cannot all be attributed to OSA rather than to the coexistence of the two conditions or to risk factors shared by both. The design of this review therefore permits a descriptive comparison of OS with COPD alone, but not an estimate of the cardiovascular burden attributable to OSA.

Several factors may account for the heterogeneity observed across studies, including population characteristics, diagnostic criteria for OSA (different apnea‐hypopnea index thresholds were applied, and not all studies used PSG as the diagnostic modality), and cardiovascular outcome definitions. Additionally, one study excluded patients with severe cardiovascular diseases, such as uncontrolled hypertension or acute heart failure, which may have contributed to an underestimation of cardiovascular disease prevalence. Another study relied on self‐reported physician‐diagnosed COPD and OSA, which may have introduced misclassification bias.

### 4.4. Future Directions and Clinical Relevance

Future prospective studies with cardiovascular disease as a predefined outcome and standardized diagnostic criteria are needed to better characterize the cardiovascular burden associated with OS and to identify high‐risk phenotypes.

By highlighting the potential additional cardiovascular burden associated with OS, this review underscores the need for cardiovascular screening strategies in patients with COPD‐OSA overlap syndrome.

Additionally, future studies should ideally include three comparison groups—OS, COPD alone, and OSA alone—with cardiovascular disease prespecified as an adjusted outcome, to clarify the independent and additive contributions of each condition.

## 5. Conclusions

This systematic review summarizes the available evidence on the prevalence of cardiovascular diseases in patients with OS compared with those with COPD alone. The majority of included studies reported a higher prevalence of cardiovascular diseases, especially arterial hypertension, in the OS group, frequently reaching statistical significance in unadjusted comparisons; however, because no study adjusted for key confounders such as age, BMI, and smoking, these associations remain hypothesis‐generating.

The interpretation of all these findings is limited because of the heterogeneity across studies, the observational design, and the fact that the cardiovascular disease was reported as baseline characteristics, not as predefined outcomes.

Despite these limitations, the available evidence suggests that patients with OS may represent a subgroup with an increased cardiovascular burden. These findings highlight the potential importance of systematic cardiovascular assessment in patients with COPD and suspected or confirmed OSA.

Well‐designed prospective studies with cardiovascular disease as a predefined outcome are needed to clarify the true cardiovascular burden associated with OS.

Including the “hypoxic burden” in future studies for assessing specific OSA‐related cardiovascular risk has been proposed as a potential predictor of cardiovascular outcomes in patients with OS.

A new patient‐centered approach is needed in the management of COPD, starting from the very first clinical visit, with a focus on identifying cardiovascular comorbidities that may influence the onset of exacerbations and the evolution of COPD. The growing need for intervention guidelines, for the early and active screening of cardiovascular diseases in patients with COPD and OS, and the implementation of targeted therapy have opened new avenues for research.

**Nomenclature**
AF: atrial fibrillation
AHI: apnea‐hypopnea index
BMI: body mass index
CAD: coronary artery disease
COPD: chronic obstructive pulmonary disease
CPAP: continuous positive airway pressure
FEV1: forced expiratory volume in 1 second
FVC: forced vital capacity
GOLD: Global Initiative for Chronic Obstructive Lung Disease
HF: heart failure
HSAT: home sleep apnea testing
HTN: hypertension
ICD: International Classification of Diseases
ICD‐10: International Classification of Diseases‐10
ICD‐9: International Classification of Diseases‐9
iNH: isolated nocturnal hypoxia
NIV: noninvasive ventilation
NR: not reported
OS: overlap syndrome (COPD and OSA)
OSA: obstructive sleep apnea
PAP: pulmonary artery pressure
PECO: Population, Exposure, Comparator, Outcome
PRISMA: Preferred Reporting Items for Systematic Reviews and Meta‐Analyses
PSG: polysomnography
REM: rapid eye movement
RP: respiratory polygraphy

## Author Contributions

Conceptualization: S.F.O. and C.C.D. Methodology: S.F.O. and S.D.R. Software: S.F.O. Validation: S.F.O., S.D.R., and C.C.D. Formal analysis: S.F.O. Investigation: S.F.O., S.D.R. Resources: S.F.O, and S.D.R. Data curation: S.F.O. and S.D.R. Writing—original draft preparation: S.F.O. Writing—review and editing: S.F.O., S.D.R. and C.C.D. Visualization: S.F.O. Supervision: C.C.D.

## Funding

No funding was received for this manuscript.

## Disclosure

The authors have reviewed and edited the output and take full responsibility for the content of this publication. All authors have read and agreed to the published version of the manuscript.

## Ethics Statement

The authors have nothing to report.

## Consent

The authors have nothing to report.

## Conflicts of Interest

The authors declare no conflicts of interest.

## Supporting information


**Supporting Information** Additional supporting information can be found online in the Supporting Information section. The following supporting material is available for this article: Table S1: PRISMA checklist; Table S2: complete search strategies for PubMed and Web of Science; Table S3: structure of the data extraction table; Table S4: risk‐of‐bias assessment tools and Newcastle–Ottawa Scale scores of the included studies; Table S5: excluded studies and reasons for exclusion; Table S6: study population characteristics of the included studies; Table S7: prevalence of cardiovascular disease in the included studies.

## Data Availability

Data sharing not applicable—no new data generated, or the article describes entirely theoretical research.
