# Supplementary material for: Prevalence of Cardiovascular Diseases in COPD–OSA Overlap Syndrome Versus COPD Alone: A Systematic Review
Source: Pulm Med. 2026 Sep 12;2026:2311043. doi: 10.1155/pm/2311043 (PMC13570493; doi:10.1155/pm/2311043)
Supplement: Supplementary file 1 [file PM-2026-2311043-s001.docx]

**Table S1.** *PRISMA Checklist*

| **Section and Topic** | **Item #** | **Checklist item** | **Location where item is reported** |
| --- | --- | --- | --- |
| **TITLE** | | |  |
| Title | 1 | Identify the report as a systematic review. | Page 1 |
| **ABSTRACT** | | |  |
| Abstract | 2 | See the PRISMA 2020 for Abstracts checklist. | Page 1 |
| **INTRODUCTION** | | |  |
| Rationale | 3 | Describe the rationale for the review in the context of existing knowledge. | Page 1-2 |
| Objectives | 4 | Provide an explicit statement of the objective(s) or question(s) the review addresses. | Page 1-2 |
| **METHODS** | | |  |
| Eligibility criteria | 5 | Specify the inclusion and exclusion criteria for the review and how studies were grouped for the syntheses. | Page 2, Section 2.2 |
| Information sources | 6 | Specify all databases, registers, websites, organisations, reference lists and other sources searched or consulted to identify studies. Specify the date when each source was last searched or consulted. | Page 2, Section 2.3 |
| Search strategy | 7 | Present the full search strategies for all databases, registers and websites, including any filters and limits used. | Page 3,  Section 2.3, Table S2 |
| Selection process | 8 | Specify the methods used to decide whether a study met the inclusion criteria of the review, including how many reviewers screened each record and each report retrieved, whether they worked independently, and if applicable, details of automation tools used in the process. | Page 3, Section 2.4 |
| Data collection process | 9 | Specify the methods used to collect data from reports, including how many reviewers collected data from each report, whether they worked independently, any processes for obtaining or confirming data from study investigators, and if applicable, details of automation tools used in the process. | Page 3, Section 2.4 |
| Data items | 10a | List and define all outcomes for which data were sought. Specify whether all results that were compatible with each outcome domain in each study were sought (e.g. for all measures, time points, analyses), and if not, the methods used to decide which results to collect. | Page 3, Section 2.4 |
|  | 10b | List and define all other variables for which data were sought (e.g. participant and intervention characteristics, funding sources). Describe any assumptions made about any missing or unclear information. | Page 3, Section 2.4 |
| Study risk of bias assessment | 11 | Specify the methods used to assess risk of bias in the included studies, including details of the tool(s) used, how many reviewers assessed each study and whether they worked independently, and if applicable, details of automation tools used in the process. | Page 3-4, Section 2.5 |
| Effect measures | 12 | Specify for each outcome the effect measure(s) (e.g. risk ratio, mean difference) used in the synthesis or presentation of results. | Page 4,  Section 2.6 |
| Synthesis methods | 13a | Describe the processes used to decide which studies were eligible for each synthesis (e.g. tabulating the study intervention characteristics and comparing against the planned groups for each synthesis (item #5)). | Page 4,  Section 2.6 |
|  | 13b | Describe any methods required to prepare the data for presentation or synthesis, such as handling of missing summary statistics, or data conversions. | Page 3-4,  Section 2.4,2.6 |
|  | 13c | Describe any methods used to tabulate or visually display results of individual studies and syntheses. | Page 4,  Section 2.6 |
|  | 13d | Describe any methods used to synthesize results and provide a rationale for the choice(s). If meta-analysis was performed, describe the model(s), method(s) to identify the presence and extent of statistical heterogeneity, and software package(s) used. | Page 4,  Section 2.6 |
|  | 13e | Describe any methods used to explore possible causes of heterogeneity among study results (e.g. subgroup analysis, meta-regression). | Page 4,  Section 2.6 |
|  | 13f | Describe any sensitivity analyses conducted to assess robustness of the synthesized results. | Page 4,  Section 2.6 |
| Reporting bias assessment | 14 | Describe any methods used to assess risk of bias due to missing results in a synthesis (arising from reporting biases). | Page 4,  Section 2.6 |
| Certainty assessment | 15 | Describe any methods used to assess certainty (or confidence) in the body of evidence for an outcome. | Page 4,  Section 2.6 |
| **RESULTS** | | |  |
| Study selection | 16a | Describe the results of the search and selection process, from the number of records identified in the search to the number of studies included in the review, ideally using a flow diagram. | Page 4,  Section 3.1 |
|  | 16b | Cite studies that might appear to meet the inclusion criteria, but which were excluded, and explain why they were excluded. | Page 4,  Section 3.1, Table S5 |
| Study characteristics | 17 | Cite each included study and present its characteristics. | Page 4, Section 3.2 |
| Risk of bias in studies | 18 | Present assessments of risk of bias for each included study. | Table S6 |
| Results of individual studies | 19 | For all outcomes, present, for each study: (a) summary statistics for each group (where appropriate) and (b) an effect estimate and its precision (e.g. confidence/credible interval), ideally using structured tables or plots. | Page 5, Section 3.3,  Table 1 |
| Results of syntheses | 20a | For each synthesis, briefly summarise the characteristics and risk of bias among contributing studies. | Page 5-9,  Section 3.4 |
|  | 20b | Present results of all statistical syntheses conducted. If meta-analysis was done, present for each the summary estimate and its precision (e.g. confidence/credible interval) and measures of statistical heterogeneity. If comparing groups, describe the direction of the effect. | Page 5-9,  Section 3.4 |
|  | 20c | Present results of all investigations of possible causes of heterogeneity among study results. | Page 11,  Section 4.3 |
|  | 20d | Present results of all sensitivity analyses conducted to assess the robustness of the synthesized results. | Page 4,  Section 3.4 |
| Reporting biases | 21 | Present assessments of risk of bias due to missing results (arising from reporting biases) for each synthesis assessed. | Page 4, Section 2.6 |
| Certainty of evidence | 22 | Present assessments of certainty (or confidence) in the body of evidence for each outcome assessed. | Page 4, Section 2.6 |
| **DISCUSSION** | | |  |
| Discussion | 23a | Provide a general interpretation of the results in the context of other evidence. | Page 9-10, Section 4.1 |
|  | 23b | Discuss any limitations of the evidence included in the review. | Page 11, Section 4.3 |
|  | 23c | Discuss any limitations of the review processes used. | Page 11, Section 4.3 |
|  | 23d | Discuss implications of the results for practice, policy, and future research. | Page 11,  Section 4.4 |
| **OTHER INFORMATION** | | |  |
| Registration and protocol | 24a | Provide registration information for the review, including register name and registration number, or state that the review was not registered. | Page 2,  Section 2.1 |
|  | 24b | Indicate where the review protocol can be accessed, or state that a protocol was not prepared. | Page 2,  Section 2.1 |
|  | 24c | Describe and explain any amendments to information provided at registration or in the protocol. | Page 2,  Section 2.1 |
| Support | 25 | Describe sources of financial or non-financial support for the review, and the role of the funders or sponsors in the review. | Page 12 |
| Competing interests | 26 | Declare any competing interests of review authors. | Page 12 |
| Availability of data, code and other materials | 27 | Report which of the following are publicly available and where they can be found: template data collection forms; data extracted from included studies; data used for all analyses; analytic code; any other materials used in the review. | Data extraction tables, study characteristics, and additional supporting material are available in the Supplementary section |

*From:*  Page MJ, McKenzie JE, Bossuyt PM, Boutron I, Hoffmann TC, Mulrow CD, et al. The PRISMA 2020 statement: an updated guideline for reporting systematic reviews. BMJ 2021;372:n71. doi: 10.1136/bmj.n71. This work is licensed under CC BY 4.0. To view a copy of this license, visit <https://creativecommons.org/licenses/by/4.0/>

**Table S2.** Search strings

The complete search strings used for both primary and secondary search are as follows:

| Web of Science: | First search: |
| --- | --- |
|  | TS=(("chronic obstructive pulmonary disease" OR COPD) AND ("obstructive sleep apnea" OR OSA OR "sleep apnea syndrome")) |
|  | Second search: |
|  | TS=(("chronic obstructive pulmonary disease" OR COPD) AND ("obstructive sleep apnea" OR OSA OR "sleep apnea syndrome") AND ( "cardiovascular disease" OR hypertension OR "coronary artery disease" OR "heart failure" OR arrhythmia* OR "atrial fibrillation" OR stroke OR "cerebrovascular disorder*")) |
| PubMed: | First search: |
|  | ("Pulmonary Disease, Chronic Obstructive"[MeSH] OR COPD[tiab] OR "chronic obstructive pulmonary disease"[tiab] ) AND ( "Sleep Apnea, Obstructive"[MeSH] OR OSA[tiab] OR "obstructive sleep apnea"[tiab] OR "sleep apnea syndrome"[tiab] ) |
|  | Second search: |
|  | ("Pulmonary Disease, Chronic Obstructive"[MeSH] OR COPD[tiab] OR "chronic obstructive pulmonary disease"[tiab] ) AND ( "Sleep Apnea, Obstructive"[MeSH] OR OSA[tiab] OR "obstructive sleep apnea"[tiab] OR "sleep apnea syndrome"[tiab] ) AND ( "Cardiovascular Diseases"[MeSH] OR "cardiovascular disease"[tiab] OR hypertension[tiab] OR "Hypertension"[MeSH] OR "Coronary Artery Disease"[MeSH] OR "coronary artery disease"[tiab] OR "heart failure"[tiab] OR "Heart Failure"[MeSH] OR arrhythmia*[tiab] OR "Atrial Fibrillation"[MeSH] OR stroke[tiab] OR "Cerebrovascular Disorders"[MeSH]) |

**Table S3.** *Head of the extraction table*

| Author | Year | Country | Study design | Data source | Total sample size | COPD + OSA (n) | COPD alone (n) | Mean age | % Male | Mean BMI / Obesity | OSA definition |
| --- | --- | --- | --- | --- | --- | --- | --- | --- | --- | --- | --- |
| HTN OS n (%) | **HTN COPD alone n (%)** | **CAD OS n (%)** | **CAD COPD alone n (%)** | **HF OS n (%)** | **HF COPD alone n (%)** | **AF OS n (%)** | **AF COPD alone n (%)** | **Stroke OS n (%)** | **Stroke COPD alone n (%)** | **CVD OS n (%)** | **CVD COPD alone n (%)** |
| Adjusted analysis (Yes/No) | | **Confounders adjusted for** | | **Baseline or follow-up data** | | **Notes** | **Results** |  |  |  |  |

Abbreviations: AF-Atrial Fibrillation, BMI-Body Mass Index, CAD-Coronary Artery Disease, COPD-Chronic Obstructive Pulmonary Disease, CVD-Cardiovascular Disease, HF -Heart Failure, HTN-Hypertension, n-number, OSA-Obstructive Sleep Apnea, OS-Overlap Syndrome (COPD and OSA).

**Table S4.** *Risk of bias Assessment tools.*

| Author | Year | Study design | Data source | Score NOS | Quality category |
| --- | --- | --- | --- | --- | --- |
| Marin et al. | 2025 | Prospective observational cohort | CHAIN cohort | 8 | High |
| Coiffier et al. | 2024 | Prospective observational cohort | e-Meuse study (clinical cohort) | 7 | High |
| Nguyen et al. | 2024 | Retrospective observational cohort | Single-center hospital cohort | 9 | High |
| Hansson et al. | 2023 | Cross-sectional study | Single-center hospital cohort | 6 | Moderate |
| Zeng et al. | 2022 | Cross-sectional study | Hospital-based multicenter cohort (4 tertiary hospitals) | 6 | Moderate |
| Zhang et al. | 2022 | Cross-sectional study | Single-center hospital cohort | 7 | High |
| Climaco et al. | 2022 | Cross-sectional study | Hospital-based multicenter cohort (3 centers) | 6 | Moderate |
| Tang et al. | 2021 | Cross-sectional study | Single-center hospital records | 7 | High |
| Zhao et al. | 2021 | Cross-sectional study | Single-center hospital cohort | 7 | High |
| Liu et al. | 2021 | Retrospective observational cohort | Single-center hospital records | 5 | Moderate |
| Tang et al. | 2021 | Retrospective cohort study | Single-center hospital cohort | 9 | High |
| Wang et al. | 2020 | Cross-sectional study | Hospital-based outpatient population | 7 | High |
| Hu et al. | 2020 | Cross-sectional study | Single-center hospital cohort | 7 | High |
| Sun et al. | 2019 | Cross-sectional study | Single-center hospital cohort | 7 | High |
| Wang et al. | 2019 | Cross-sectional study | Single-center hospital cohort | 6 | Moderate |
| Zhu et al. | 2019 | Multicenter cross-sectional study | Hospital-based multicenter cohort | 9 | High |
| Fitzgibbons et al | 2019 | Cross-sectional study | Single-center hospital cohort/VABoston Healthcare System | 5 | Moderate |
| Du et al | 2018 | Cross-sectional study | NHANES 2005-2008 | 6 | Moderate |
| Steveling et al | 2014 | Cross-sectional study | Outpatient pulmonary clinics (2 centers) | 8 | High |

Abbreviations: NOS – Newcastle-Ottawa Score

**Table S5.** *Excluded studies and reasons for exclusion.*

|  | Authors | Year | Title | Reasons |
| --- | --- | --- | --- | --- |
| 1 | Hansen et al. | 2025 | Examining the impact of obstructive sleep apnea on cognitive function in severe COPD | Wrong outcome. No cardiovascular outcome |
| 2 | Prakash et al. | 2025 | Proportion of sleep-related breathing disorders and their association with echocardiographic parameters in stable patients with chronic obstructive pulmonary disease: a cross-sectional observational exploratory study | Wrong outcome. No cardiovascular outcome |
| 3 | Zhang et al. | 2025 | The value of cough sound monitoring via an audio-enabled smartwatch for OSA screening in COPD patients: a cross-sectional exploratory study | Wrong outcome. No comparative data |
| 4 | Wang et al. | 2025 | FFMI: A Pivotal Indicator Bridging Pulmonary, Sleep, and Systemic Factors in COPD-OSA Overlap Patients | Wrong outcome. Cardiovascular disease excluded by design. |
| 5 | Camargo PF et al. | 2025 | Impact of obstructive sleep apnea on functional performance and muscle quality of patients with COPD | Wrong outcome. No cardiovascular outcome |
| 6 | Pham et al. | 2025 | Incidence of arrhythmias in chronic obstructive pulmonary disease, obstructive sleep apnea, and overlap syndrome: A retrospective cohort study | Wrong outcome. No cardiovascular outcome. Incidence data |
| 7 | Kim et al. | 2024 | Prevalence of and factors associated with likely obstructive sleep apnea in individuals with airflow limitation | Wrong diagnosis of OSA |
| 8 | Le et al. | 2024 | Prevalence of obstructive sleep apnea in patients with chronic obstructive pulmonary disease in Vietnam | Wrong outcome. No cardiovascular outcome |
| 9 | Yang et al. | 2024 | The role of neutrophil to lymphocyte ratio in patients with COPD-OSA overlap syndrome | Wrong outcome. No cardiovascular outcome |
| 10 | De la Fuente et al. | 2024 | The overlap of chronic obstructive pulmonary disease and obstructive sleep apnea in hospitalizations for acute exacerbation of chronic obstructive pulmonary disease | Wrong population. Acute COPD exacerbation. No cardiovascular outcomes primary or secondary |
| 11 | Silva et al. | 2024 | Validation of a Portable Respiratory Monitoring System for the Diagnosis of Obstructive Sleep Apnea in Patients with Chronic Obstructive Pulmonary Disease: A Crossectional Study | Wrong outcome. No cardiovascular outcome |
| 12 | Liu et al. | 2024 | Exploring the impact of OSA on short-term survival in patients with AECOPD admitted to the ICU | Wrong population. Acute COPD exacerbation |
| 13 | Zhu et al. | 2024 | Evaluation of nocturnal apnea and airflow limitation as indicators for cognitive dysfunction in patients with chronic obstructive pulmonary disease/obstructive sleep apnea hypopnea syndrome overlap syndrome | Wrong outcome. No cardiovascular outcome |
| 14 | Hu et al. | 2024 | The clinical and hemodynamic characteristics of pulmonary hypertension in patients with OS | Wrong outcome. Outcome not relevant to cardiovascular comorbidity prevalence; study focuses on pulmonary hypertension in a selected population. |
| 15 | Ianosi et al. | 2024 | Insights into the Overlap of Chronic Obstructive Pulmonary Disease and Sleep Apnea: Experience from the Clinic of Pneumology, Târgu Mures | Wrong population. No COPD comparator group |
| 16 | de Censo et al. | 2024 | Postural balance in COPD with obstructive sleep apnoea: a cross-sectional study | Wrong outcome. No cardiovascular outcome |
| 17 | Kang et al. | 2024 | Estimating the prevalence and clinical significance of chronic obstructive pulmonary disease-obstructive sleep apnea overlap in South Korea | Wrong diagnosis of OSA |
| 18 | Yeh et al. | 2024 | Outcomes of coronary artery bypass grafting (CABG) in patients with OSA-COPD overlap syndrome versus COPD alone: an analysis of US Nationwide Inpatient Sample | Wrong outcome. No cardiovascular outcome |
| 19 | Peng et al. | 2023 | A risk-predictive model for obstructive sleep apnea in patients with chronic obstructive pulmonary disease | Wrong outcome. Cardiovascular outcomes not assessed as study outcomes. |
| 20 | Camargo et al. | 2023 | Obstructive sleep apnea reduces functional capacity and impairs cardiac autonomic modulation during submaximal exercise in patients with chronic obstructive pulmonary disease: A follow-up study | Wrong outcome. No cardiovascular outcome |
| 21 | Camargo et al. | 2023 | Impact of Obstructive Sleep Apnea on Cardiac Autonomic Control during the Respiratory Sinus Arrhythmia Maneuver in Patients with Chronic Obstructive Pulmonary Disease | Wrong outcome. No cardiovascular outcome |
| 22 | Sanchez-Azofra et al. | 2023 | Inflammation biomarkers in OSA, chronic obstructive pulmonary disease, and chronic obstructive pulmonary disease/OSA overlap syndrome | Wrong outcome. No cardiovascular outcome |
| 23 | Kaddah et al. | 2023 | Predictors of obstructive sleep apnea in patients with chronic obstructive pulmonary disease | Wrong outcome. No cardiovascular outcome |
| 24 | Hashiguchi et al. | 2023 | Interaction of BMI and respiratory status in obstructive sleep apnea, a cross-sectional COPD study | Wrong outcome. Cardiovascular disease excluded by design. No COPD comparator group |
| 25 | Donovan et al. | 2023 | Sleep Testing and Mortality in a Propensity-matched Cohort of Patients with Chronic Obstructive Pulmonary Disease | Wrong population. No OS |
| 26 | Kaya et al. | 2023 | Determination and Comparison of Sleep Quality and Sleep Disorders in Patients with COPD According to GOLD Groups | Foreign language |
| 27 | Shvaiko LI et al. | 2023 | The combination of chronic respiratory diseases with obstructive sleep apnoea syndrome in clean-up workers of Chornobyl NPP accident | Wrong outcome. No cardiovascular outcome |
| 28 | Channick et al. | 2022 | Effects of Obstructive Sleep Apnea and Obesity on 30-Day Readmissions in Patients with Chronic Obstructive Pulmonary Disease A Cross-Sectional Mediation Analysis | Wrong outcome. No cardiovascular outcome |
| 29 | Kendzerska et al. | 2022 | Coexistence of clinically significant obstructive sleep apnea with physician-diagnosed asthma or chronic obstructive pulmonary disease: A population study of prevalence and mortality | Wrong outcome. No cardiovascular outcome |
| 30 | Marin-Oto et al. | 2022 | Soluble RAGE in COPD, with or without coexisting obstructive sleep apnoea | Wrong outcome. No cardiovascular outcome |
| 31 | Silva Climaco et al. | 2022 | Sleep quality in COPD patients: correlation with disease severity and health status | Wrong outcome. No cardiovascular outcome. Cardiovascular comorbidities not assessed as primary or secondary outcomes. |
| 32 | Wollsching-Strobel et al. | 2022 | The Impact of Non-Invasive Ventilation on Sleep Quality in COPD Patients | Wrong outcome. No cardiovascular outcome. No COPD comparator group |
| 33 | Alkhayat et al. | 2021 | Prevalence of obstructive sleep apnea in patients with chronic obstructive pulmonary disease | Wrong outcome. No cardiovascular outcome |
| 34 | Nattusami et al. | 2021 | Co-existing obstructive sleep apnea among patients with chronic obstructive pulmonary disease | Wrong outcome. No cardiovascular outcome |
| 35 | Mohammad et al. | 2021 | Prevalence of obstructive sleep apnea among patients with chronic obstructive pulmonary disease | Wrong outcome. No cardiovascular outcome |
| 36 | Bae et al. | 2021 | Mortality prediction in chronic obstructive pulmonary disease and obstructive sleep apnea | Wrong outcome. Cardiovascular diseases not assessed as prevalence outcome |
| 37 | Luehrs et al. | 2021 | Cognitive performance is lower among individuals with overlap syndrome than in individuals with COPD or obstructive sleep apnea alone: association with carotid artery stiffness | Wrong outcome. No cardiovascular outcome |
| 38 | Sami et al. | 2021 | Polysomnography findings of patients with overlap syndrome according to severity of lower airway obstruction | Wrong outcome. No cardiovascular outcome. No OS comparator group |
| 39 | Zhou et al. | 2020 | Metabolic syndrome prevalence in patients with obstructive sleep apnea syndrome and chronic obstructive pulmonary disease: Relationship with systemic inflammation | Wrong outcome. No cardiovascular outcome. just cardiovascular risk |
| 40 | Ioachimescu et al. | 2020 | Obstructive Lung Disease and Obstructive Sleep Apnea (OLDOSA) cohort study: 10-year assessment | Wrong outcome. No cardiovascular outcome |
| 41 | Soares de Carvalho Junior et al. | 2020 | Overlap syndrome: the coexistence of OSA further impairs cardiorespiratory fitness in COPD | Wrong outcome. No cardiovascular outcome |
| 42 | Zhang et al. | 2020 | Moderate-to-Severe Obstructive Sleep Apnea and Cognitive Function Impairment in Patients with COPD | Wrong outcome. No cardiovascular outcome |
| 43 | Yang et al. | 2020 | The Bronchiectasis in COPD-OSA Overlap Syndrome Patients | Wrong outcome. No cardiovascular outcome |
| 44 | Wu et al. | 2020 | Pulmonary Function Influences the Performance of Berlin Questionnaire, Modified Berlin Questionnaire, and STOP-Bang Score for Screening Obstructive Sleep Apnea in Subjects with Chronic Obstructive Pulmonary Disease | Wrong outcome. Cardiovascular comorbidities were not reported in sufficient detail or as a predefined outcome. |
| 45 | Channick et al. | 2020 | Effects of Obstructive Sleep Apnea and Obesity on Thirty-Day Readmissions in Patients with Chronic Obstructive Pulmonary Disease | Wrong outcome. No cardiovascular outcome |
| 46 | Hong et al. | 2020 | The Economic Burden of Comorbid Obstructive Sleep Apnea Among Patients with Chronic Obstructive Pulmonary Disease | Wrong outcome. No cardiovascular outcome |
| 47 | Masoud et al. | 2020 | Long-term adherence to home mechanical ventilation: a 10-year retrospective, single-centre cohort study | Wrong population. No COPD comparator group. No OS comparator group |
| 48 | Shah et al. | 2020 | Sleep Quality and Nocturnal Symptoms in a Community-Based COPD Cohort | Wrong diagnosis of OSA. Just risk factors of OSA. |
| 49 | Naranjo et al. | 2020 | Undiagnosed OSA May Significantly Affect Outcomes in Adults Admitted for COPD in an Inner-City Hospital | Wrong outcome. Cardiovascular diseases not assessed as prevalence outcome. Acute COPD exacerbation |
| 50 | Cantero C et al. | 2020 | Long-Term Noninvasive Ventilation in the Geneva Lake Area: Indications, Prevalence, and Modalities | Wrong population. No COPD comparator group. No OS comparator group |
| 51 | Li et al. | 2019 | Impact of Lung Function and SDB on Incident Myocardial Infarction and Heart Failure: A Community-based Study | Wrong population. No clear definition of COPD |
| 52 | Zhang et al. | 2019 | Obstructive Sleep Apnea in Patients With Fibrotic Interstitial Lung Disease and COPD | Wrong outcome. No cardiovascular outcome |
| 53 | Xiong et al. | 2019 | The Screening Value Of ESS, SACS, BQ, And SBQ On Obstructive Sleep Apnea In Patients With Chronic Obstructive Pulmonary Disease | Wrong outcome. Cardiovascular diseases not assessed as prevalence outcome |
| 54 | Holmedahl et al. | 2019 | Validation of peripheral arterial tonometry as tool for sleep assessment in chronic obstructive pulmonary disease | Wrong outcome. No cardiovascular outcome. No comparative data. No OS group |
| 55 | Latshang TD et al. | 2019 | Sleep and breathing disturbances in patients with chronic obstructive pulmonary disease traveling to altitude: a randomized trial | Wrong population. No COPD comparator group. No OS comparator group |
| 56 | Maestri R et al. | 2019 | Physiological and clinical characteristics of patients with COPD admitted to an inpatient pulmonary rehabilitation program: A real-life study | Wrong outcome. Cardiovascular outcomes not assessed as study outcomes |
| 57 | Kendzerska et al. | 2019 | Cardiovascular Outcomes and All-Cause Mortality in Patients with Obstructive Sleep Apnea and Chronic Obstructive Pulmonary Disease (Overlap Syndrome) | Wrong population. No COPD alone, just COPD with AHI<30/h |
| 58 | Lopes et al. | 2018 | Chronic obstructive pulmonary disease severity and its association with obstructive sleep apnea syndrome: impact on cardiac autonomic modulation and functional capacity | Wrong outcome. No cardiovascular outcome |
| 59 | Archontogeorgis et al. | 2018 | Mean Platelet Volume and Platelet Distribution Width in Patients With Obstructive Sleep Apnea Syndrome and Concurrent Chronic Obstructive Pulmonary Disease | Wrong outcome. No cardiovascular outcome |
| 60 | Schreiber et al. | 2018 | Prevalence and Predictors of Obstructive Sleep Apnea in Patients with Chronic Obstructive Pulmonary Disease Undergoing Inpatient Pulmonary Rehabilitation | Wrong outcome. No COPD comparator group. No OS comparator group. Does not report specific cardiovascular disease prevalence separately for OS versus COPD alone |
| 61 | Macrea et al. | 2018 | The peripheral neutrophils in subjects with COPD-OSA overlap syndrome and severe comorbidities: A feasible inflammatory biomarker? | Wrong outcome. No cardiovascular outcome |
| 62 | Gunduz et al. | 2018 | Prevalence of overlap syndrome in chronic obstructive pulmonary disease patients without sleep apnea symptoms | Wrong outcome. No cardiovascular outcome |
| 63 | Jimborean et al. | 2018 | Association Between Chronic Obstructive Pulmonary Disease and Sleep Apnea - Overlap Syndrome-Experience of Pulmonology Clinic Tg. Mures, Romania | Wrong population. No clear definition of OS (COPD/OSA/OHS) |
| 64 | Hirayama et al. | 2018 | Association of obstructive sleep apnoea with acute severity of chronic obstructive pulmonary disease exacerbation: a population-based study | Wrong outcome. Cardiovascular outcomes not assessed as study outcomes |
| 65 | Mungo Pissulin et al. | 2018 | The triad of obstructive sleep apnea syndrome, COPD, and obesity: sensitivity of sleep scales and respiratory questionnaires | Wrong outcome. No cardiovascular outcome |
| 66 | Jonassen et al. | 2018 | Associations between obstructive lung disease and symptoms of obstructive sleep apnoea in a general population | Wrong population. No COPD comparator group |
| 67 | Xie et al. | 2018 | Prevalence of pulmonary embolism in patients with obstructive sleep apnea and chronic obstructive pulmonary disease: The overlap syndrome | Wrong population. No COPD alone |
| 68 | Choi et al. | 2017 | Overlap syndrome of COPD and OSA in Koreans | Wrong population. No OS. No cardiovascular outcome |
| 69 | Soler et al. | 2017 | Age, gender, neck circumference, and Epworth sleepiness scale do not predict obstructive sleep apnea (OSA) in moderate to severe chronic obstructive pulmonary disease (COPD): The challenge to predict OSA in advanced COPD | Wrong outcome. No cardiovascular outcome |
| 70 | Janocko et al. | 2017 | Obstructive sleep apnea, asthma and chronic obstructive pulmonary disease-long-term outcomes in a cohort of 1,314 veterans | Wrong outcome. No cardiovascular outcome |
| 71 | Andres-Blanco et al. | 2017 | Assessment of automated analysis of portable oximetry as a screening test for moderate-to-severe sleep apnea in patients with chronic obstructive pulmonary disease | Wrong outcome. No cardiovascular outcome |
| 72 | He et al. | 2017 | Coexistence of OSA may compensate for sleep related reduction in neural respiratory drive in patients with COPD | Wrong outcome. No cardiovascular outcome |
| 73 | Silva JLR Júnior et al. | 2017 | Sleep-disordered breathing in patients with COPD and mild hypoxemia: prevalence and predictive variables | Wrong outcome. Cardiovascular comorbidities not assessed as primary or secondary outcomes. |
| 74 | Matsumoto et al. | 2016 | Microalbuminuria in Patients with Obstructive Sleep Apnea-Chronic Obstructive Pulmonary Disease Overlap Syndrome | Wrong outcome. No cardiovascular outcome. Small COPD group |
| 75 | Taranto-Montemurro et al. | 2016 | Cardiac Sympathetic Hyperactivity in Patients with Chronic Obstructive Pulmonary Disease and Obstructive Sleep Apnea | Wrong outcome. Cardiovascular diseases not assessed as prevalence outcome |
| 76 | Dragonieri et al. | 2016 | Exhaled breath profiling in patients with COPD and OSA overlap syndrome: a pilot study | Wrong outcome. No cardiovascular outcome |
| 77 | Krachman et al. | 2016 | Effect of Emphysema Severity on the Apnea-Hypopnea Index in Smokers with Obstructive Sleep Apnea | Wrong population. No COPD comparator group |
| 78 | Jen et al. | 2016 | Evaluation Of Right Ventricular Remodeling And Expansion Of Extracellular Volume (ecv) Using Cardiac Magnetic Resonance Imaging In Co-Existent Chronic Obstructive Pulmonary Disease And Obstructive Sleep Apnea | Wrong outcome. No cardiovascular outcome |
| 79 | Huang et al. | 2016 | Analysis of airway resistance and hypoxemia in overlap syndrome | Wrong outcome. No cardiovascular outcome |
| 80 | Tamai K et al. | 2016 | Nocturnal Oxygen Desaturation Index is Inversely Correlated with Airflow Limitation in Patients with Chronic Obstructive Pulmonary Disease | Wrong population. No overlap comparator group |
| 81 | Zhao YY et al. | 2016 | Sleep Apnea and Obstructive Airway Disease in Older Men: Outcomes of Sleep Disorders in Older Men Study | Wrong population. No clear diagnosis of COPD. No cardiovascular outcome |
| 82 | Soler et al. | 2015 | High Prevalence of Obstructive Sleep Apnea in Patients with Moderate to Severe Chronic Obstructive Pulmonary Disease | Wrong outcome. No cardiovascular outcome |
| 83 | Wang et al. | 2015 | Associated bone mineral density and obstructive sleep apnea in chronic obstructive pulmonary disease | Wrong outcome. No cardiovascular outcome |
| 84 | Gothi et al. | 2015 | Impact of overlap syndrome on severity of acute exacerbation of chronic obstructive pulmonary disease | Wrong outcome. No cardiovascular outcome |
| 85 | Wang et al. | 2015 | Obstructive sleep apnea exacerbates airway inflammation in patients with chronic obstructive pulmonary disease | Wrong outcome. No cardiovascular outcome |
| 86 | Turcani et al. | 2015 | The prevalence of obstructive sleep apnea in patients hospitalized for COPD exacerbation | Wrong population. Acute COPD exacerbation |
| 87 | Faria et al. | 2015 | Sleep Apnea Clinical Score, Berlin Questionnaire, or Epworth Sleepiness Scale: which is the best obstructive sleep apnea predictor in patients with COPD? | Wrong outcome. No cardiovascular outcome |
| 88 | Pahwa P et al. | 2015 | A community-based participatory research methodology to address, redress, and reassess disparities in respiratory health among First Nations | Wrong population. No COPD comparator group. No OS comparator group |
| 89 | Scott et al. | 2014 | Examination of pulse oximetry tracings to detect obstructive sleep apnea in patients with advanced chronic obstructive pulmonary disease | Wrong population. No OS |
| 90 | Badr et al. | 2014 | Lipoprotein-associated phospholipase A2 levels as a predictor of cardiovascular risks in patients with COPD and obstructive sleep apnea | Wrong outcome. No cardiovascular outcome |
| 91 | Greenberg-Dotan et al. | 2014 | Increased prevalence of obstructive lung disease in patients with obstructive sleep apnea | Wrong population (OSA-based cohort, not COPD-based) |
| 92 | Azuma M et al. | 2014 | Associations among chronic obstructive pulmonary disease and sleep-disordered breathing in an urban male working population in Japan | Wrong outcome. No cardiovascular outcome |
| 93 | Ganga et al. | 2014 | Risk of new-onset atrial fibrillation in elderly patients with the overlap syndrome: a retrospective cohort study | Wrong outcome. No cardiovascular outcome. Incidence data |
| 94 | Lacedonia et al. | 2013 | Daytime PaO2 in OSAS, COPD and the combination of the two (overlap syndrome) | Wrong outcome. No cardiovascular outcome |
| 95 | Stanchina et al. | 2013 | Impact of CPAP Use and Age on Mortality in Patients with Combined COPD and Obstructive Sleep Apnea: The Overlap Syndrome | Wrong outcome. No cardiovascular outcome |
| 96 | Nural et al. | 2013 | Inflammatory Processes and Effects of Continuous Positive Airway Pressure (CPAP) in Overlap Syndrome | Wrong outcome. No cardiovascular outcome |
| 97 | Wang TY et al. | 2013 | Nocturnal CPAP improves walking capacity in COPD patients with obstructive sleep apnoea | Wrong outcome. Cardiovascular comorbidities not assessed as primary or secondary outcomes. |
| 98 | Ardic et al. | 2012 | Chronic obstructive pulmonary disease and obstructive sleep apnea symptoms: an outpatient-based population study in Turkey | Wrong diagnosis of OSA. Just risk factors of OSA |
| 99 | Pasquina et al. | 2012 | What Does Built-In Software of Home Ventilators Tell Us? An Observational Study of 150 Patients on Home Ventilation | Wrong outcome. No cardiovascular outcome |
| 100 | Diomidous M et al. | 2012 | The Syndrome of Sleep Apnea in the Elderly Suffering from COPD and Live in the County of Attica, Greece | Wrong diagnosis of OSA. Just risk factors of OSA |
| 101 | Marin et al. | 2010 | Outcomes in Patients with Chronic Obstructive Pulmonary Disease and Obstructive Sleep Apnea The Overlap Syndrome | Wrong outcome. No cardiovascular outcome |
| 102 | Shaya et al. | 2009 | Elevated economic burden in obstructive lung disease patients with concomitant sleep apnea syndrome | Wrong outcome. No cardiovascular outcome |
| 103 | Lopez-Acevedo et al. | 2009 | Overlap syndrome: an indication for sleep studies? A pilot study | Wrong outcome. No cardiovascular outcome |
| 104 | Mermigis et al. | 2006 | Health-related quality of life in patients with obstructive sleep apnea syndrome and COPD (overlap syndrome) | Wrong outcome. No cardiovascular outcome |
| 105 | Bednarek et al. | 2005 | There is no relationship between chronic obstructive pulmonary disease and obstructive sleep apnea syndrome: A population study | Wrong outcome. No cardiovascular outcome |
| 106 | Sanders MH et al. | 2003 | Sleep and sleep-disordered breathing in adults with predominantly mild obstructive airway disease | Wrong population. No COPD comparator group |
| 107 | Resta O et al. | 2002 | Hypercapnia in overlap syndrome: possible determinant factors | Wrong outcome. No cardiovascular outcome |

Abbreviations: COPD-Chronic Obstructive Pulmonary Disease, OHS- Obesity Hypoventilation Syndrome, OSA-Obstructive Sleep Apnea, OS-Overlap Syndrome (COPD and OSA).

**Table S6.** *Study population characteristics*

| Study ID | Total sample size | COPD + OSA (n) | COPD alone (n) | Mean age (years) | % Male | Mean BMI (kg/m2)/ Obesity data | OSA definition |
| --- | --- | --- | --- | --- | --- | --- | --- |
| Marin et al.  2025 | 428 | 136 | 177 | OS ~ 68;  COPD~65 | OS 83%, COPD 73% | OS 29.7; COPD 26.4 | HSAT, AHI ≥ 15/h |
| Coiffier et al.  2024 | 308 | 95 | 105 | OS 65; COPD: 65 (median) | OS 65%; COPD 58% | OS 31; COPD 24 (median) | RP or PSG |
| Nguyen et al.  2024 | 168 | 44 | 124 | OS 70.6; COPD 71.6 (median) | OS 54.5%; COPD 49.2% | OS 34; COPD 23.5 | Physician diagnostic or Previous sleep study with AHI ≥10 |
| Hansson et al.  2023 | 105 | 49 | 56 | OS 70.1 ± 8.2; COPD 66.3 ± 9.5 | OS 55.1%; COPD 33.9% | OS 30.0 ± 6.4; COPD 26.4 ± 6.7; Obesity (BMI ≥30): 52% vs 30.9% | HSAT, AHI ≥ 15/h |
| Zeng et al.  2022 | 886 | 264 | 622 | OS 67.8 ± 9.6; COPD 68.1 ± 8.5 | OS 92.0%; COPD 85.2%; | OS 25.1 ± 4.6; COPD 23.4 ± 4.0; | PSG, AHI ≥ 15/h |
| Zhang et al.  2022 | 842 | 194 | 648 | OS 62 ± 7;  COPD 63 ± 8 | OS 72.68; COPD 69.91 | OS 24 ± 4; COPD 22 ± 4 | PSG, AHI ≥ 15/h |
| Climaco et al.  2022 | 102 | 51 | 51 | OS 69.6 ± 10.7;  COPD 66.2 ± 9.2 | OS 73%, COPD 47% | OS 26.3 ± 4.6; COPD 25.7 ± 5.3 | PSG, AHI ≥ 15/h |
| Tang et al.  2021 | 518 | 74 | 222 | OS 60.95±9.35;  COPD 60.76 ± 9.30 | OS 70%;  COPD 70% | OS 26.89 ± 3.39; COPD 22.53 ± 3.76 | PSG, AHI ≥ 5/h + symptoms |
| Zhao et al.  2021 | 252 | 180 | 72 | OS 64.7; COPD 65.7 (median) | OS 69%; COPD 60% | OS 25.8 ; COPD 24.2 (median) | PSG, AHI ≥ 5/h |
| Liu et al.  2021 | 206 | 120 | 86 | OS 70.07 ± 8.66; COPD 68.31 ± 7.51 | OS 73.3%; COPD 74.4% | OS 28.95 ± 2.96; COPD 23.84 ± 4.06 | RP, AHI ≥5/h + symptoms or AHI ≥15/h |
| Tang et al.  2021 | 395 | 79 | 158 | OS 60.7 ± 9.6;  COPD 60.6 ± 9.5 | OS 69.6% COPD 69.6% | OS 26.5 ± 2.3; COPD 24.4 ± 7.1 | PSG, AHI ≥5/h |
| Wang et al.  2020 | 277 | 106 | 171 | OS 67.7 ± 10.6;  COPD 66.3 ± 11.2 | OS: 84.9%; COPD 85.4% | OS 29.0 ± 3.3; COPD 26.4 ± 3.5 | PSG, AHI ≥ 15/h |
| Hu et al.  2020 | 968 | 660 | 308 | OS 65.79 ± 9.69; COPD 65.45 ± 9.24; | OS 84.9%; COPD 83.1% | OS 23.68 ± 4.40; COPD 22.41 ± 4.04; | PSG, AHI ≥ 5/h |
| Sun et al.  2019 | 106 | 56 | 50 | OS 69.9 ± 10.9; COPD 69.1 ± 9.2 | OS 92.9%; COPD 90.0% | OS 24.14; COPD 23.37 | RP, AHI ≥10/h |
| Wang et al.  2019 | 95 | 25 | 25 | OS 64.1 ± 4.0;  COPD 63.2 ± 3.4 | OS: 76%; COPD: 64% | OS 28.1 ± 2.0; COPD 27.1 ± 1.5 | PSG, AHI ≥ 15/h |
| Zhu et al.  2019 | 766 | 520 | 246 | OS 65.6 ± 10.0; COPD 65.0 ± 9.4 | OS 84.8%; COPD 84.1% | OS 23.8 ± 4.5; COPD 22.3 ± 3.9 | PSG, AHI ≥ 5/h |
| Fitzgibbons et al.  2019 | 256 | 61 | 195 | OS 69.3 ± 8.83;  COPD 71.1 ± 8.28 | OS 100%; COPD 97.9% | OS 33.2; COPD 27.1 (median)/obesity 75.4% vs 26.2% | Self-reported physician diagnosed OSA |
| Du et al.  2018 | 10388 | 90 | 695 | OS 55.8 ± 1.5;  COPD 53.9 ± 0.7 | OS 49.4%; COPD 36.9% | Obesity OS 69.7%; COPD 38.0% | Self-reported physician diagnosed OSA |
| Steveling et al.  2014 | 177 | 33 | 144 | OS 65.6 (9.6);  COPD 63.8 ± 8.8 | OS 88%  COPD 58% | OS 30.0; COPD 24.2 (median) | RP, AHI >10/h |

Abbreviations: AHI-Apnea-Hypopnea Index, COPD-Chronic Obstructive Pulmonary Disease, n-number, HSAT-Home Sleep Apnea Testing, OSA-Obstructive Sleep Apnea, OS-Overlap Syndrome (COPD and OSA), PSG- Polysomnography, RP- Respiratory Polygraphy.

**Table S7.** *Prevalence of cardiovascular disease*

| Study ID | HTN overlap n (%) | HTN COPD alone n (%) | CAD overlap n (%) | CAD COPD alone n (%) | HF overlap n (%) | HF COPD alone n (%) | AF overlap n (%) | AF COPD alone n (%) | Stroke overlap n (%) | Stroke COPD alone n (%) | CVD overlap | CVD COPD alone |
| --- | --- | --- | --- | --- | --- | --- | --- | --- | --- | --- | --- | --- |
| Marin et al. 2025 | 58 (43%) | 61 (34%) | 19 (14%) | 18 (10%) | NR | NR | NR | NR | NR | NR | NR | NR |
| Coiffier et al. 2024 | 51 (54%) | 32 (30%) | 26 (27%) | 15 (14%) | NR | NR | NR | NR | 8 (8.4%) | 5 (4.8%) | 69 (73%) | 52 (50%) |
| Nguyen et al. 2024 | 33 (75%) | 62 (50.0%) | 9 (20.5%) | 28 (22.6%) | 14 (31.8%) | 27 (21.8%) | 11 (25%) | 22 (17.7%) | 4 (9.1%) | 7 (5.6%) | NR | NR |
| Hansson et al. 2023 | 35 (71.4%) | 25 (44.6%) | 12 (24.5%) | 5 (8.9%) | 10 (20.4%) | 4 (7.1%) | 5 (10.2%) | 3 (5.4%) | 1 (2.0%) | 9 (16.1%) | NR | NR |
| Zeng et al. 2022 | 126 (47.7%) | 254 (40.8%) | 43 (16.3%) | 126 (20.3%) | NR | NR | NR | NR | 21 (8.0%) | 39 (6.3%) | NR | NR |
| Zhang et al. 2022 | 93 (47.9%) | 190 (29.3%) | 20 (10.3%) | 67 (10.3%) | NR | NR | NR | NR | NR | NR | NR | NR |
| Climaco et al. 2022 | 17 (33.3%) | 23 (45.1%) | NR | NR | NR | NR | NR | NR | NR | NR | NR | NR |
| Tang et al. 2021 | 45 (60.8%) | 66 (29.7%) | 19 (25.7%) | 26 (11.7%) | 8 (10.8%) | 3 (1.4%) | 8 (10.8%) | 12 (5.4%) | 15 (20.3%) | 23 (10.4%) | NR | NR |
| Zhao et al. 2021 | 74 (41%) | 15 (21%) | 25 (14%) | 3 (4%) | NR | NR | NR | NR | NR | NR | NR | NR |
| Liu et al. 2021 | 62 (51.7%) | 41 (47.7%) | 55 (45.8%) | 38 (44.2%) | NR | NR | NR | NR | NR | NR | NR | NR |
| Tang et al. 2021 | 56 (70.8%) | 45 (28.5%) | 24 (30.4%) | 15 (9.5%) | 28 (35.4%) | 14 (8.9%) | 11 (13.9%) | 7 (4.4%) | 15 (19.0%) | 21 (13.3%) | NR | NR |
| Wang et al. 2020 | 71 (67.0%) | 111 (64.9%) | 64 (60.4%) | 76 (44.4%) | NR | NR | NR | NR | NR | NR | NR | NR |
| Hu et al. 2020 | 270 (40.91%) | 98 (31.81%) | NR | NR | NR | NR | 17 (2.58%) | 7 (2.27%) | 63 (9.55%) | 28 (9.09%) | 161 (24.39%) | 66 (21.43%) |
| Sun et al. 2019 | 24 (42.9%) | 23 (46.0%) | 13 (23.2%) | 7 (14.0%) | 9 (16.1%) | 6 (12.0%) | NR | NR | cerebrovascular 6 (10.7%) | cerebrovascular 3 (6%) | NR | NR |
| Wang et al. 2019 | 15 (60%) | 9 (36%) | 9 (36%) | 4 (16%) | NR | NR | NR | NR | 5 (20%) | 2 (8%) | NR | NR |
| Zhu et al. 2019 | 219 (42.1%) | 86 (35.0%) | NR | NR | NR | NR | NR | NR | NR | NR | NR | NR |
| Fitzgibbons et al 2019 | 44 (72.1%) | 109 (55.9%) | 24 (39.3%) | 49 (25.1%) | 13 (21.3%) | 13 (6.7%) | NR | NR | NR | NR | NR | NR |
| Du et al. 2018 | 63 (60.6%) | 324 (41.1%) | NR | NR | NR | NR | NR | NR | NR | NR | 47 (46%) | 188 (21.8%) |
| Steveling et al. 2014 | 20 (61%) | 54 (38%) | NR | NR | NR | NR | NR | NR | NR | NR | 6 (18%) | 18 (13%) |

Abbreviations: AF-Atrial Fibrillation, AHI – Apnea-Hypopnea Index, BMI-Body Mass Index, CAD-Coronary Artery Disease, COPD-Chronic Obstructive Pulmonary Disease, CVD-Cardiovascular Disease, HF -Heart Failure, HTN-Hypertension, n-number, OSA-Obstructive Sleep Apnea, OS-Overlap Syndrome (COPD and OSA
